# Population genetic analysis of a medicinally significant Australian rainforest tree, *Fontainea picrosperma* C.T. White (Euphorbiaceae): biogeographic patterns and implications for species domestication and plantation establishment

**DOI:** 10.1186/s12870-016-0743-2

**Published:** 2016-02-29

**Authors:** R. W. Lamont, G. C. Conroy, P. Reddell, S. M. Ogbourne

**Affiliations:** GeneCology Research Centre, Faculty of Science, Health, Engineering and Education, University of the Sunshine Coast, Maroochydore DC, Queensland 4558 Australia; EcoBiotics Ltd., Yungaburra, Queensland 4884 Australia

**Keywords:** Biodiscovery, Cancer, EBC-46, Population genetics, Rainforest refugia, Wet Tropics

## Abstract

**Background:**

*Fontainea picrosperma*, a subcanopy tree endemic to the rainforests of northeastern Australia, is of medicinal significance following the discovery of the novel anti-cancer natural product, EBC-46. Laboratory synthesis of EBC-46 is unlikely to be commercially feasible and consequently production of the molecule is via isolation from *F. picrosperma* grown in plantations.

Successful domestication and plantation production requires an intimate knowledge of a taxon’s life-history attributes and genetic architecture, not only to ensure the maximum capture of genetic diversity from wild source populations, but also to minimise the risk of a detrimental loss in genetic diversity via founder effects during subsequent breeding programs designed to enhance commercially significant agronomic traits.

**Results:**

Here we report the use of eleven microsatellite loci (*PIC* = 0.429; *P*_*ID*_ = 1.72 × 10^−6^) to investigate the partitioning of genetic diversity within and among seven natural populations of *F. picrosperma*. Genetic variation among individuals and within populations was found to be relatively low (*A* = 2.831; *H*_*E*_ = 0.407), although there was marked differentiation among populations (*PhiPT* = 0.248). Bayesian, UPGMA and principal coordinates analyses detected three main genotypic clusters (*K* = 3), which were present at all seven populations. Despite low levels of historical gene flow (*N*_*m*_ = 1.382), inbreeding was negligible (*F* = -0.003); presumably due to the taxon’s dioecious breeding system.

**Conclusion:**

The data suggests that *F. picrosperma* was previously more continuously distributed, but that rainforest contraction and expansion in response to glacial-interglacial cycles, together with significant anthropogenic effects have resulted in significant fragmentation. This research provides important tools to support plantation establishment, selection and genetic improvement of this medicinally significant Australian rainforest species.

**Electronic supplementary material:**

The online version of this article (doi:10.1186/s12870-016-0743-2) contains supplementary material, which is available to authorized users.

## Background

Of the more than 1000 drugs of novel chemical structure (New Chemical Entities) approved for use by international regulatory authorities between 1981 and 2010; greater than 60 % were derived from natural products [12]. This is unsurprising as almost 3 billion years of evolution has created comprehensive libraries of natural product small molecule ligands, targeted to interact with specific macromolecules [43]. The chemical complexity and functional diversity of these natural secondary metabolites has not been fully explored and continues to provide a significant resource for the potential discovery of new pharmaceuticals. As a consequence, the conservation of biodiversity for the discovery of novel natural compounds has significant social and economic value [2, 18, 50].

Australia is one of a small number of countries that are considered ‘mega-diverse’, which combined occupy only 10 % of the Earth’s surface, yet support over 70 % of the world’s biodiversity [40]. The tropical rainforests of Queensland are inscribed on UNESCO’s World Heritage list and contain a substantial proportion of Australia’s rainforest biota. As such, they are generally recognised as one of the continent’s main hotspots of biodiversity [10, 25, 51] with high levels of endemism due to ~35 million years of geographic isolation and considerable climatic change during the Tertiary [13, 26]. *Fontainea picrosperma* C.T. White (family Euphorbiaceae), a dioecious subcanopy tree endemic to Queensland’s tropical rainforests, illustrates the opportunity for continuing discovery of novel pharmaceuticals from nature and the value in protecting Australia’s mega-diverse rainforest flora.

*F. picrosperma* is of substantial current interest following the discovery of a novel epoxy-tigliane (EBC-46) with putative anti-cancer activity, in this species [4]. EBC-46 is a potent activator of protein kinase C and a single intra-lesional injection into solid tumours has been shown to result in rapid ablation and cure of tumours in pre-clinical murine models [4]. At present, EBC-46 is under development for use as both a human and a veterinary pharmaceutical and has entered a first-in-man Phase I clinical trial in Australia (ACTRN12614000685617; http://www.anzctr.org.au). EBC-46 cannot currently be produced by laboratory synthesis on a commercial scale and instead is manufactured for research, preclinical and clinical use by purification from plantation-grown material of *F. picrosperma*.

A more detailed knowledge of the ecology and genetics of this promising species will be critical to its domestication and future commercial drug production from plantations. Acquiring a basic knowledge of the species chromosome structure, such as chromosome number and levels of ploidy will be of future value. However, gaining an understanding of the genetic diversity and structure of natural populations, patterns of gene flow, and the taxon’s mating system is particularly important [6, 39]. For instance, artificial populations of outcrossing dioecious species such as *F. picrosperma* may be particularly vulnerable to a loss of reproductive fitness arising from inbreeding among similar genotypes situated in close proximity, or departures from random mating due to the disproportionate contributions of particular individuals to fertilisation events, leading to reduced vigour [9, 39].

In this study, we investigate the population genetic structure within and among natural stands of *F. picrosperma* from across the natural geographic range of this species. Our aim was to assess the relevance of populations within the context of the species as a whole, whilst simultaneously maximising the capture of available genetic variation from wild individuals. Furthermore, by ensuring maximal genetic diversity in crosses designed to enhance commercially significant agronomic traits, the microsatellite-based technique will provide an important management tool to support subsequent breeding programs used to develop *F. picrosperma* as a niche tree crop for the commercial supply of EBC-46.

## Results

### Genetic diversity

Despite an initial screening of 65 labelled microsatellite primer pairs, only 11 moderately polymorphic loci (mean *PIC* = 0.429) were found suitable for the analysis of population genetic diversity and structure in *F. picrosperma* (Table 1); the remaining 54 loci were monomorphic. A total of 37 alleles were resolved in the 218 individuals analysed, with between two and seven alleles per locus (Table 1) and a mean number of alleles per locus (*A*) of 2.831 (Table 2). Following correction for population size differences, the mean population level measure of allelic richness (*A*_*R*_) decreased to 2.480 alleles per locus (Table 2). A total of seven private alleles (*A*_*P*_) were detected within five of the seven populations surveyed. In the east, two were detected in the large, putatively refugial population at Boonjie (*n* = 45) and one at Topaz (*n* = 22), while another was resolved in the 17 individuals sampled at Malanda in the central portion of the species distribution. A further three unique alleles were detected in the western populations of East Barron (*A*_*P*_ = 2; *n* = 26) and Evelyn Highlands (*A*_*P*_ = 1; *n* = 68). Proportional representations of private allelic richness for each population following rarefaction (*PA*_*R*_) are given in Table 2.

**Table 1:** Characterization of eleven microsatellite loci isolated from 218 individuals of *Fontainea picrosperma*

Locus GenBank	Repeat motif	Primer sequences (5′–3′)	Size range (bp)	*PIC*	*N*_*A*_	*H*_*O*_	*H*_*E*_	*F*_*IS*_
FP21 KC759358	(TA)_13_	F: TCACTGAATTCGCTTGGTTG R: TGCAAATACCAGAAGTGCCA	194–204	0.596	6	0.532	0.664	0.000
FP32 KC759359	(GT)_8_	F: CTGGCTTGCATTTGCTTGTA R: TGCTAAACTTCAAGGGCTTAGG	190–192	0.329	2	0.339	0.416	−0.034
FP39 KC759362	(GA)_15_	F: CTGCACGACAAGAAAACTCG R: TGAGTCAATATTGTAAGGGAATTATGA	203–213	0.293	3	0.280	0.325	−0.004
FP40 KC759363	(TG)_16_	F: TTCTCGTCCTCTACTGGGCT R: CCCTACCTTTCCCACTCACA	134–152	0.455	6	0.550	0.551	−0.096
FP44 KC759364	(AT)_7_	F: TGAAGCTAATTGCTTGATCTTCC R: GGGTATTTATTTTCTTGTTTGTTTCC	112–122	0.390	5	0.459	0.505	−0.117
FP47 KC759365	(TC)_7_	F: CCTAAAAGTGCCCTTTGGCTA R: TGTGACTTTCCATGCTCCAG	238–242	0.284	3	0.307	0.338	−0.192
FP49 KM213753	(GA)_8_	F: TTTATACAACCACCAGTCGCC R: CACCTTCACTGAAATTCTCTTCTTC	171–175	0.479	3	0.468	0.537	0.013
FP56 KM213754	(TA)_14_	F: CAGGGCTTAGAATCGGGTGT R: TCACATCCTAGGTCCGTTCAC	258–270	0.776	7	0.391	0.806	0.390
FP59 KM213755	(AT)_11_	F: TCCCTCCTGTTAAGACTGTTACA R: CCTTCACCATCAATCAGCCG	210–218	0.163	2	0.143	0.179	0.128
FP62 KM213756	(TC)_11_	F: TGAAAATGCTGACCAAATATGTGA R: AGTTTCCCAGGATCCCACAT	271–273	0.375	2	0.468	0.501	−0.086
FP64 KM213757	(GAC)_11_	F: ACGGTGAAGACGATGATGGT R: CGTGTGTTACCTCTTCTTCAGC	108–129	0.581	6	0.385	0.631	0.075
			Mean	0.429	4.1	0.393	0.496	0.007

**Fig. 1:**
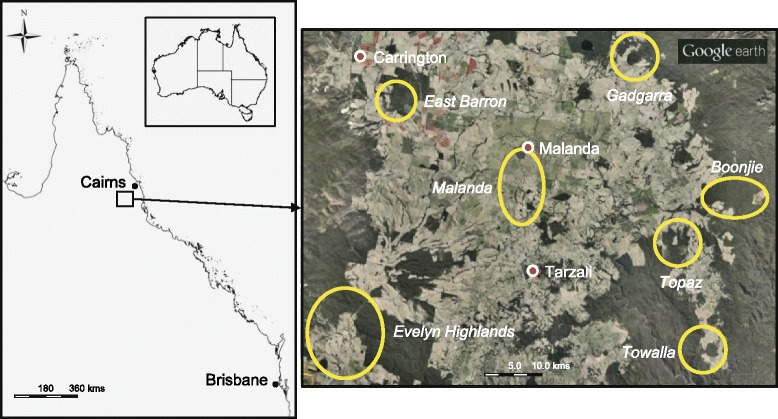
Map of sampling locations for *F. picrosperma* genetic variation study. Each sampling area is represented by a yellow circle or oval

Samples were collected from the Atherton Tablelands, Australia from seven locations shown in Fig. 1. *PIC* polymorphic information content; *N*_*A*_ number of alleles; *H*_*O*_ observed heterozygosity; *H*_*E*_ expected heterozygosity; *F*_*IS*_ inbreeding coefficient

**Table 2:** Summary of genetic measures for the 218 individuals sampled from seven populations of *F. picrosperma*

Population	*n*	*n♀*	*n♂*	*A*	*A*_*R*_	*PA*_*R*_	*H*_*O*_	*H*_*E*_	*F*
Evelyn Highlands	68	5	15	3.182	2.580	0.060	0.350	0.432	0.149
Boonjie	45	19	9	3.545	2.840	0.120	0.459	0.507	0.066
East Barron	26	3	12	2.909	2.440	0.180	0.374	0.410	0.078
Malanda	17	10	1	2.636	2.500	0.050	0.487	0.447	−0.115
Topaz	22	11	5	3.000	2.650	0.110	0.417	0.415	0.015
Gadgarra	18	4	5	2.182	1.980	0.000	0.298	0.264	−0.139
Towalla	22	3	8	2.364	2.240	0.000	0.397	0.372	−0.093
Mean	31.03 (1.974)	55	55	2.831 (0.142)	2.480 (0.108)	0.076 (0.026)	0.397 (0.023)	0.407 (0.022)	−0.003 (0.030)

*n*, number of plants sampled per population; n♀, number of female plants sampled per population; n♂, number of male plants sampled per population; *A*, mean number of alleles per locus; *A*_*R*_, allelic richness (based on a minimal sample size of 17); *PA*_*R*_, private allelic richness; *H*_*O*_ mean observed heterozygosity; *H*_*E*_ mean expected heterozygosity; *F* fixation index. Standard errors in parenthesis

Measures of observed heterozygosity (*H*_*O*_) were relatively low across populations, ranging from 0.298–0.487 (mean *H*_*O*_ *=* 0.397) and were more or less concordant with levels of expected heterozygosity (*H*_*E*_) (0.264 to 0.507; mean *H*_*E*_ = 0.407) calculated under conditions of Hardy-Weinberg Equilibrium (HWE) (Table 2). Consequently, combined populations of the dioecious *F. picrosperma* displayed an overall negligible level of inbreeding (mean *F* = −0.003), however individual population values ranged between *F* = −0.139 to 0.149, indicating a low to moderate excess of either heterozygotes or homozygotes at particular sites (Table 2).

Although the level of genetic diversity resolved in the 218 samples tested was reasonably low, the statistical confidence for individual identification using the 11 loci employed in this study was quite high (*P*_*ID*_ = 1.72 × 10^−6^) with only two individuals from East Barron found to share the same multilocus genotype. The other 216 samples had unique multilocus genotypes. Several microsatellite markers displaying minimal polymorphism (2–3 alleles; Table 1) were removed from the analysis to assess its sensitivity to a reduction (and by inference, increase) in loci; whilst there was minimal impact on fundamental genetic diversity outputs, a considerable proportion of the discriminatory power to accurately identify individuals was lost. The validation of the ability to discriminate individuals using the complete set of 11 markers identified for this study is therefore significant with regards to future selection and breeding programs.

### Population structure and gene flow

Analysis of Molecular Variance (AMOVA) found most (75 %) of the species diversity to reside within populations, with the rest of the variation due to differences between populations (*PhiPT* = 0.248, *p* = 0.001) (Additional file 1: Table S2; supporting information). Wright’s *F*-statistics further subdivided population differentiation into a combination of differences among individuals (*F*_*IS*_ = 0.096) and populations (*F*_*ST*_ = 0.153, *p* = 0.001), translating to a low to moderate level of historical gene flow (mean *N*_*m*_ = 1.382 individuals/generation), sufficient to prevent or slow the rate of genetic drift between sites (Table 3). Pairwise population *F*_*ST*_ values were all significantly different from zero (*p* <0.001) and ranged from a level of minimal differentiation (*F*_*ST*_ = 0.035; *N*_*m*_ = 6.880) between the relatively proximate populations at Topaz and Towalla to a maximum distance (*F*_*ST*_ = 0.302; *N*_*m*_ = 0.579) between the two northern, most isolated populations, Gadgarra and East Barron (Table 3; Fig. 1). In fact, apart from a low level of contact suggesting Boonjie as the possible source population (Boonjie-Gadgarra *N*_*m*_ = 1.585; Boonjie-East Barron *N*_*m*_ = 1.360), neither Gadgarra nor East Barron displayed sufficient gene flow (*N*_*m*_ < 1.000) with any of the other populations to prevent genetic drift [52]. Conversely, both Evelyn Highlands and Boonjie displayed evidence of genetic exchange with most other populations, supporting the hypothesis that both of these populations may be long term refugia.

**Table 3:** Pairwise population *F*_*ST*_ (below diagonal) and *N*_*m*_ (above diagonal) values

	Evelyn Highlands	Boonjie	East Barron	Malanda	Topaz	Gadgarra	Towalla
Evelyn Highlands	0.000	**2.122**	0.850	**2.677**	**1.985**	0.956	**1.291**
Boonjie	0.105	0.000	**1.360**	**2.125**	**2.911**	**1.585**	**1.859**
East Barron	0.227	0.155	0.000	0.979	0.786	0.579	0.812
Malanda	0.085	0.105	0.203	0.000	**3.672**	0.682	**3.104**
Topaz	0.112	0.079	0.241	0.064	0.000	0.834	**6.880**
Gadgarra	0.207	0.136	0.302	0.268	0.231	0.000	0.745
Towalla	0.162	0.119	0.235	0.075	0.035	0.251	0.000

Mean *F*_*ST*_ = 0.153. Mean *N*_*m*_ = 1.382. Effective levels of past gene flow among the seven populations of *F. picrosperma* assessed are indicated in bold type. Values based on 999 permutations

**Fig. 2:**
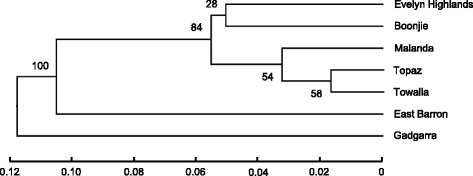
UPGMA cluster analysis of the seven populations of *F. picrosperma.* Genetic distances were calculated using pairwise *F*_*ST*_ [58] measures of genetic distance

**Fig. 3:**
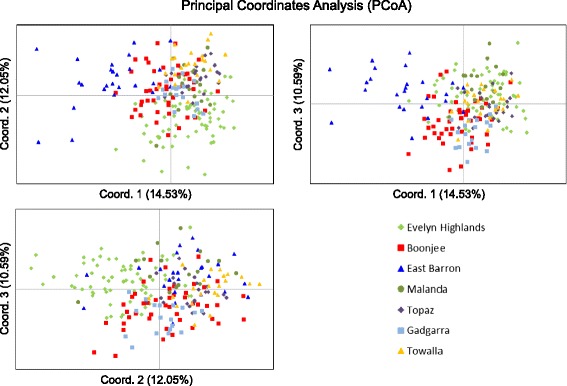
Principal coordinates analysis (PCoA) of *F. picrosperma* individuals using genetic distance matrices. Individuals from the seven populations are indicated by the symbols illustrated. Coordinate axis 1 accounts for 14.53 % of variation within the data, axis 2, 12.05 % and axis 3, 10.59 %. The cumulative percentage for the first three axes combined explain 37.17 % of the variation

**Fig. 4:**
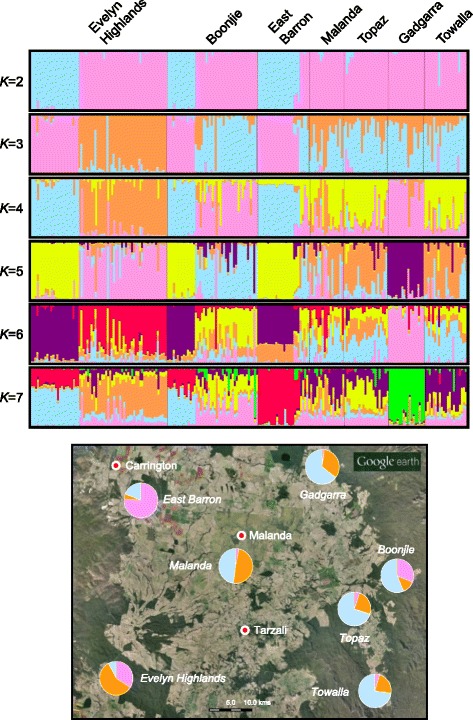
Admixture bar plots representing the identity of individuals based on assignment using Bayesian modelling. Each individual is shown as a vertical line partitioned into *K* coloured segments whose length is proportional to the individual coefficients of membership in *K* = 2 to *K* = 7 genetic clusters that represent the populations assessed (top). The average membership of individuals of the *K* = 3 clusters (selected as the best estimate of the number of genetic clusters following implementation of the Evanno method [17]) for each sub-population are presented as pie charts, superimposed onto the location map to provide geographic perspective (bottom)

The UPGMA cluster analysis (Fig. 2) further confirmed Gadgarra and East Barron as more divergent, with approximately 88 % and 90 % similarity, respectively, to the remaining populations of the species (Fig. 2). The most western and eastern peripheral populations of Evelyn Highlands (on and around Mt. Hypipamee, 1125 m asl) and Boonjie (on the western slopes of Mt Bartle Frere, 1622 m asl) displayed a moderate gene flow (*N*_*m*_ = 2.122) strongly suggesting that similarity (*F*_*ST*_ = 0.105) may be linked to their putative status as long-term interglacial refugia (Figs. 2, 3 and 4; Table 3), rather than recent gene flow per se.

Principal coordinates analysis (PCoA) detected a close genetic relationship between individuals within populations due to low levels of diversity (Fig. 3). The first three principal components were the main axes of variation as indicated by the scree plot (Additional file 2: Figure S1) and broken stick analysis according to Jackson [27], explaining a cumulative variation between individuals of only 37.17 %. The scree plot indicated a gradual decay in eigenvalues rather than a steep decline, further highlighting the low levels of diversity and genetic structure observed in this species. However, despite failing to clearly separate populations into discrete clusters, the PCoA analysis mostly concurred with the UPGMA cluster analysis and supports our hypothesis of the existence of three main groups; a western group (Evelyn Highlands), an eastern group (Boonjie) and a central group (Topaz, Towalla, Malanda and Gadgarra). For example, individuals from Boonjie and Evelyn Highlands form two separate but genetically overlapping groups that combined overlap the majority of individuals from Topaz, Towalla, Malanda and Gadgarra, which themselves cluster tightly together. Although Gadgarra clustered with the central populations, it seems to be somewhat inbred and genetically divergent from this group, containing a depauperate subset of the genetic variation found within the central populations (Table 2). In contrast, East Barron’s genetic distinctiveness was likely due to a relatively high proportion of private alleles (Table 2) and random founder effects that took place during its establishment (Figs. 2, 3 and 4). Results of the STRUCTURE analysis indicated that *ln* likelihoods of the data plateaued quickly from *K* = 3 to *K* = 4 (Additional file 3: Table S1, supporting information). Hence, *K* = 3 was selected as the best estimate of the number of genetic clusters following implementation of the Evanno et al. [17] method in STRUCTURE HARVESTER. However, additional genetic structure of biological relevance at different levels of K is also apparent (Fig. 4). While each of the three genetic clusters was present at the seven sites assessed, proportions differed substantially among the populations. Calculation of the average proportionality of each genetic cluster for each population support the UPGMA and PCoA analyses and the presence of three main groups of *F. picrosperma* (Fig. 4). For example, the average proportionality of each genetic cluster for the western group (Evelyn Highlands) was approximately 34 % K_1_ (pink), 58 % K_2_ (orange) and 8 % K_3_ (blue), compared to 31 % K_1_, 13 % K_2_ and 56 % K_3_ for the eastern group (Boonjie) and 4 % K_1_, 32 % K_2_ and 64 % K_3_ for the central group (Topaz, Towalla, Malanda and Gadgarra). Evelyn Highlands and Boonjie therefore have a more uniform but differing spread of the three genetic clusters as compared to the central group, while one of the genetic clusters that is strongly represented in both Evelyn Highlands and Boonjie (K_1_) is only minimally represented in the plateau group, together providing support to their assignment as putative refugial populations. The Mantel test found that the geographic structuring of *F. picrosperma*’s genetic variation did not follow a predictable pattern, and no relationship was detected between genetic and geographic distance matrices among populations (*R*_*xy*_ = 0.282; *r*^*2*^ = 0.0795; *p* > 0.05).

Results of the Bottleneck analysis did not detect any signs of recent bottlenecks in five of the seven populations assessed (*p* > 0.05). However, a significant (*p* = 0.004) heterozygosity excess at ten of the eleven loci was found in both the Malanda and Gadgarra populations. This data suggests that individuals at these sites are showing effects of disruption to ‘continuous’ populations and are no longer in mutation-drift equilibrium. These effects likely reflect their long-term isolation from populations in the two putative refugial areas (Boonjie and Evelyn Highlands) for this species and may have been further exacerbated by anthropogenic activities such as aboriginal burning since the Last Glacial Maximum and large-scale rainforest clearing in more recent times.

## Discussion

There are three key findings from this study that are highly relevant not only to the domestication and breeding of *Fontainea picrosperma* for plantation production of EBC-46, but also to understanding the biogeographic history of the species. (1) The overall genetic diversity of *F. picrosperma* was relatively low but the seven populations sampled from across the natural range were genetically distinct. (2) The levels of inbreeding in the individual populations were negligible despite their current discontinuous distribution and fragmentation. (3) Within the context of the low levels of genetic diversity and weak genetic structure observed for this species, two putative long-term refugial areas were identified in the eastern (Boonjie) and western (Evelyn Highlands) parts of the natural distribution of the species, which align with the refugial rainforest areas of Bartle-Frere Uplands and western Atherton Uplands identified by Hilbert et al. [25].

### Genetic diversity

This is the first study to utilise microsatellites to examine genetic structure in the genus *Fontainea*. We investigated the levels and partitioning of genetic variation across the known range of *F. picrosperma* and found that the seven populations surveyed were genetically distinct despite having uniformly low levels of genetic diversity. This finding was not unexpected as many Australian plant species are characterised by low levels of genetic diversity, often as an adaptation to harsh environmental conditions [29, 51, 55], but also as a result of belonging to ancient lineages [45]. For instance, contrary to the accepted anthropomorphic view that a high level of genetic diversity bestows optimal evolutionary capability under conditions of environmental stress, James [29] found low levels of diversity in many successful species of Australia’s southwestern flora due to the purging of recombinational impedimenta (genetic load), allowing them to operate in harsh conditions at a highly adapted level. This counter-intuitive finding may also explain low genetic diversity in many of the ancient lineages in the Australian rainforest flora [22], including the results of this study for *F. picrosperma*. In essence, these rainforest taxa are highly adapted over long time periods to specific niches provided by the rainforest environment. As a consequence of this specialisation and niche differentiation in an essentially stable local environment, they experience only modest selection pressure during periods of climatic stability and when environmental conditions change, they retreat into the remaining environmental habitat to which they are so well adapted.

### Inbreeding

In general, the results indicate extremely low levels of inbreeding (*F* = −0.003), which despite local populations having been isolated through glacial events, and more recently by anthropogenic habitat fragmentation, would be expected in an obligate outcrossing, dioecious species like *F. picrosperma*. Even though proximate trees are likely to be siblings or half-sibs, due to the limited dispersal capabilities of *F. picrosperma*’s relatively large drupaceous fruit, this suggests that deleterious mutations may have been purged over time, as most of the diversity resolved was between individuals within populations, not among populations.

The slight excess of heterozygosity detected in some populations suggests that recent bottlenecks with subsequent founder effects due to the expansion/contraction dynamics of small populations located outside of the main refugia may be responsible for a minor degree of genetic drift causing the random fixation of alleles. However, several populations were found to exhibit an equally slight excess of homozygosity, either as a result of the lack of overall genetic variation in the species or because of consanguineous matings. Although allelic diversity was found to be low, the fact that only two individuals shared the same multilocus genotype indicates that ‘selfing’ among proximate sibs or half-sibs was of limited occurrence; in fact these two individuals may be clones. Numerous studies have found pollen travel in continuous rainforest vegetation may be within the order of several kilometres [3]; more detailed, parent-progeny research to investigate fine-scale patterns of gene flow within wild populations, aimed at maintaining optimal among production seed crops of *F. picrosperma*, is required.

### Population structure and gene flow

Stands of *F. picrosperma* occur in the upland and highland rainforests of the Atherton Tableland within a 15–20 km radius of Malanda. As such, the seven populations selected for population genetic analysis in this study likely represent a considerable proportion of the available genetic diversity within the species. It is entirely plausible that the low levels of genetic diversity and weak population structure that we have observed within *F. picrosperma* could merely be reflective of a random distribution of the diversity between individuals and populations. However, we believe that our observations reflect the existence of three distinct races or forms, including two long-term refugial races where suitable habitat is known to have persisted during less favourable times [25].

The population genetic structure of *F. picrosperma* is likely heavily influenced by the species’ life-history attributes and the effects of a long history of rainforest attrition followed by successive cycles of glacial-induced expansion and contraction upon the distribution of remaining populations. The Quaternary glacial cycles of recent geological times are known to have played a significant role in the current distributions and genetic signatures of many species [24] and based on our results this would seem to apply to *F. picrosperma*. Episodes of range expansion and contraction can have considerable genetic consequences [42] and the dynamics of the Wet Tropics rainforests corresponding to the glacial cycles of the Plio-Pleistocene are well documented [23, 33, 57]. Hence, the present-day configuration of *F. picrosperma*’s population genetic structure is likely a direct product of re-colonisation of dry sclerophyllous vegetation by tropical rainforest from refugial pockets of suitable habitat, following amelioration of the cool, dry conditions associated with past glacial cycles [8, 15, 23, 25, 33, 34, 37, 38, 51, 54, 56, 57]. It is likely that during this period several of the central populations assessed here have undergone at least some degree of geographic and genetic isolation.

The fruits of *F. picrosperma* disperse primarily by gravity with secondary long-distance dispersal facilitated either by hydrochory along drainage lines or zoochorous vectors [11, 14]. Populations therefore do not spread as a continuous wave of advance but rather are found as small and often isolated clumps or clusters, which may help to explain patterns in the geographical distribution of alleles. Nonetheless, the population genetic structure of *F. picrosperma* and the degree of historical gene flow between populations has been sufficient to maintain species’ integrity, suggesting populations were likely more continuously distributed in the past. The fact that the genus, originally described as containing a single taxon, *F. pancheri*, is composed of several highly similar taxa [21, 30], suggests vicariance due to habitat contraction occasioning genetic drift and the eventual loss of species cohesion may have been responsible for species divergences in the past.

The UPGMA cluster, principal coordinates and STRUCTURE analyses all provide a clear indication about the genetic distribution of this species. When combined with the genetic diversity analysis, the data show that the geographically distant (~28 km), peripheral populations of Boonjie and Evelyn Highlands, are genetically most diverse in comparison to the other populations whilst having elements of similarity, and form two genetically similar groups. Four of the remaining populations (Topaz, Towalla, Malanda and Gadgarra) form another genetic group, whereas the population at East Barron is genetically more divergent. We speculate that the populations at Evelyn Highlands and Boonjie represent two, genetically similar races or forms representing the two main refugial areas, where *F. picrosperma* persisted during times of sclerophyll expansion, before re-radiating out across the landscape under more favourable climatic conditions. In contrast, we suggest that the central populations of Topaz, Towalla, Malanda and Gadgarra represent a ‘plateau’ race or form that have likely expanded from small refugia during less severe climatic cycles, forming a genetically divergent race or form of *F. picrosperma*. East Barron appears to be derived from the elevated population at Evelyn Highlands (~1100 m asl), but is a genetically more divergent population, probably due to random founder effects. Gadgarra on the other hand, is genetically distinct, most likely as it contains no unique alleles and is somewhat inbred; essentially Gadgarra is a genetically depauperate variation of the plateau form. Despite the fact that the data suggests the presence of these three groups, it is important to highlight that the genetic diversity within *F. picrosperma* is low and the genetic structure between these three groups is proportionately low. In fact, the pairwise *F*_*ST*_ values between Evelyn Highlands and Boonjie, Evelyn Highlands and the plateau group, and Boonjie and the plateau group range from only 0.039 to 0.060. However, each value was significantly different from zero (*p* <0.001) and within the context of the low levels of genetic variation within this species, this is suggestive of the presence of relevant genetic structure.

It is likely that the genetic relationship between the populations can be explained not so much by linear geographic distance but by their distribution within major river catchments radiating from the putative refugial sites of Evelyn Highlands and Boonjie. However, we also recognise the possibility that our analysis could merely be reflective of a random distribution of the observed genetic diversity. Therefore, future research to test our hypothesis that two refugial races or forms and a plateau race or form of *F. picrosperma* exist will necessarily involve chloroplast DNA analysis and the sampling of additional individuals sourced along potential gene flow corridors, such as major river systems originating from the putative refugia at Evelyn Highlands and Boonjie.

### Selection, breeding, and plantation management

Knowledge of the genetic structure of source populations, mating system and patterns of gene flow are vital to the efficient establishment and management of seed orchard plantations and the production of improved open-pollinated seed [6, 7, 39]. Although the level of microsatellite variation detected in *F. picrosperma* was comparatively low, high exclusion probabilities (*P*_*ID*_) confirm that these markers will be useful in future paternity analyses and breeding programs; the former to determine patterns of gene flow in natural populations that will guide plantation design of this dioecious species, and the latter to ensure maximal genetic diversity is maintained during breeding of commercially significant agronomic traits, both of which are critical aspects of developing *F. picrosperma* as a niche tree crop for the supply of EBC-46.

Significant variation has been observed among *F. picrosperma* individuals with regard to several commercially significant agronomic traits such as growth, fruit production and EBC-46 content, suggesting that the species will be ideally suited for genetic improvement to optimise production. However, even in dioecious species, the genetic diversity of seed orchards can be eroded by a number of factors including a high proportion of ‘selfs’ arising from consanguineous matings between sibs or half-sibs, and departures from random mating due to unequal contributions of individuals to seed crops [39]. In fact, obligate outcrossing species such as *F. picrosperma* may be particularly vulnerable to losses in reproductive fitness stemming from elevated rates of inbreeding, leading to reductions in both vigour and yield [7, 35]. Therefore, to implement suitable plantation design and management options, it is necessary to have an intimate knowledge of a species’ mating system, reproductive biology, outcrossing rate and gene flow patterns in order to maximise breeding progress whilst preserving genetic diversity [5, 6, 39].

Traditionally, the most cost-effective manner of limiting inbreeding in *ex situ* populations was to position individuals in such a way that the possibility of close relatives mating would be small and hope for the best, however new techniques based on the minimisation of the global probability of consanguinity by considering the genetic relationships among trees within the entire planting have been developed [20]. Microsatellites are powerful tools for tracing pollen flow using parent/progeny arrays and work is continuing in both wild and artificial populations of *F. picrosperma* to establish which seed source and orchard variables are most likely to govern the efficiency of production plantations.

## Conclusion

*Fontainea picrosperma* is a subcanopy tree from the Atherton Tableland in Far North Queensland, Australia and is of considerable scientific and medicinal interest. The species is locally common, yet has a highly restricted range, and in relatively recent times its distribution has been heavily affected by both natural and anthropogenic habitat fragmentation. Using 11 microsatellite markers, we detected low levels of genetic diversity across the species and a population genetic structure influenced by successive cycles of glacial-induced, population expansion and contraction. The observed low levels of heterozygosity are concordant with other species of the region which have undergone similar cycles of contraction and recolonisation.

Despite the limited variation detected in this study, UPGMA cluster, Bayesian and principal coordinates analyses indicated *F. picrosperma* to be comprised of three distinct genetic races or forms. We hypothesise that these three groups broadly correspond to the existence of two long-term refugial races (Evelyn Highlands and Boonjie - on the western and eastern periphery, respectively), where suitable habitat is known to have persisted during times of eucalypt forest expansion, and an intervening plateau race that has recolonised sclerophyllous woodlands during less severe climatic cycles.

*F. picrosperma* is of significant commercial interest because it is the source plant from which the novel anti-cancer agent, EBC-46 was discovered. EBC-46 is a complex small molecule natural product that is not readily amenable to laboratory synthesis and as such, manufacture of this drug candidate will be via purification from plantation-grown raw material. Although individual specimens will be selected from the wild to establish plantations based on commercially important agronomic traits, the microsatellite-based method developed here will ensure that maximum genetic diversity is also captured. Furthermore, it will allow for careful management of future breeding programs by ensuring maximal genetic diversity in crosses designed to enhance the commercially important agronomic traits. The complex ecology and distribution patterns of this dioecious rainforest species, as well as its pharmaceutical potential, will ensure that *F. picrosperma* will be a species of significant interest into the future.

## Methods

### Study site and sample collection

*Fontainea picrosperma* occurs on soils derived from basaltic parent materials at altitudes of 700–1200 m above sea level (asl) and is restricted to an area of approximately 30 × 30 km on the southern Atherton Tableland, Queensland, Australia. Whilst it is geographically restricted and its distribution is fragmented, the species is relatively common at a local scale where suitable habitat exists.

We sampled 218 individuals from seven *F. picrosperma* populations selected to cover the geographical range of the species (Fig. 1). Leaf tissue was collected from between 17 and 68 mature plants per population, dependent upon site area and the numbers of individuals present (Table 1). The location of each individual was mapped using a handheld GPS and voucher specimens from each population have been lodged at the Queensland Herbarium (BRI). Total genomic DNA was extracted from silica-dried leaf tissue using a DNeasy™ Plant Mini Kit (Qiagen, Hilden, Germany) following the manufacturer’s instructions.

### Microsatellite analysis

A detailed description of marker development using GS-FLX Titanium chemistry (Roche Applied Science; Mannheim, Germany) is given in Agostini et al. [1]. Eleven polymorphic microsatellite loci (Table 1) with consistent PCR amplification, clear allelic variation, and clarity of electrophoretic signatures were selected to assess population genetic variation. The forward primer of each locus was direct-labelled with a fluorescent dye (VIC, PET, FAM, NED). Three multiplex PCR pools (Pool 1: *FP39*, *FP40*, *FP62*, *FP64*; Pool 2: *FP21*, *FP44*, *FP56*; Pool 3: *FP32*, *FP47*, *FP49*, *FP59*) were amplified using Multiplex PCR Plus Kits (Qiagen). Forward and reverse primers for each multiplex pool were combined in a 10× primer mix using 1–3 μM of each primer, dependent upon PCR product fluorescence intensities. Reactions, with volumes adjusted to 10 μL, each contained 1 μL of 10× primer premix, 3.0 μL of Qiagen Multiplex Buffer (2x), 3.5 μL of ddH_2_O, and 2.5 μL of template gDNA (10 ng/μL). Amplification was performed using an Eppendorf Mastercycler (Hamburg, Germany) with cycling conditions as follows: initial denaturation at 95 °C for 5 min, followed by 35 cycles of 94 °C for 30 s, 57 °C for 90 s, and 72 °C for 30 s; with a final extension at 68 °C for 10 min. PCR products were separated by capillary electrophoresis on an AB 3500 Genetic Analyser (Applied Biosystems). Fragment sizes were determined relative to an internal lane standard (GS-600 LIZ; Applied Biosystems) using GENEMARKER v. 2.4.0 (SoftGenetics LLC, PA, USA) and double-checked manually. Individuals with low or missing peaks were amplified and genotyped a second time.

### Genetic diversity

Allelic frequencies for each population were generated in GenAlEx v. 6.5 [46] and used to determine population genetic parameters including: the mean number of alleles per locus (*A*), observed heterozygosity (*H*_*O*_), unbiased genetic diversity (*H*_*E*_), and the fixation index (*F*) as a measure of past inbreeding [58]. Allelic richness (*A*_*R*_) and private allelic richness (*PA*_*R*_) for each population were obtained via rarefaction using the program HP-RARE [31] to compute the mean number of alleles per locus and the frequency of private alleles within populations, based on a minimum sample size of 17 (Malanda). Polymorphic information content (*PIC*) and probability of identity (*P*_*ID*_), i.e., the chance of individuals sharing the same multilocus genotype, was calculated in CERVUS v. 3.0.3 [32].

### Population structure and gene flow

We used a number of methods to analyse the population structure across *F. picrosperma*’s distribution. The average pair-wise level of genetic differentiation (*F*_*ST*_; [58]) between populations was determined using multi-locus comparisons in GenAlEx v. 6.5 [46] based on 999 permutations. As the *F*_*ST*_ statistic is an indirect measure of gene flow, inversely related to the effective migration rate, it was used in the following formula *N*_*m*_ = 0.25 (1- *F*_*ST*_)/*F*_*ST*_ [59] to estimate the number of migrants per generation between populations. Nei’s unbiased genetic distance (*D*; [41]) was calculated to examine patterns of genetic differentiation among populations. A hierarchical cluster analysis (UPGMA - unweighted pair group method with arithmetic averaging), using pairwise *F*_*ST*_ was performed employing 999 permutations using POPTREE2 [53]. Estimates of genetic similarity between populations were calculated from the cluster analysis.

To look for genetic relationships within and among populations, the genetic distance matrix [41] was also used in a principal coordinates analysis (PCoA; [44]). An analysis of molecular variance (AMOVA; [19]) was then applied to quantify the partitioning of genetic variation within and among populations. Both the PCoA and AMOVA were conducted using GenAlEx v. 6.5 [46]. Mantel tests [36] were used to examine the influence of between-population geographic distances on the observed patterns of genetic differentiation (Isolation by Distance; IBD) by regressing individual pairwise genetic distances against a matrix of geographic distances calculated from GPS coordinates. Levels of significance were derived from 999 random permutations using GenAlEx version 6.5 [46].

We investigated the presence of genetically differentiated groups of populations using the Bayesian genetic clustering algorithm implemented in STRUCTURE v. 2.3.4 [48]. An admixture model was applied with correlated allele frequencies and ten independent runs for each value of *K* (number of clusters) between 2 and 7 were performed, employing a burn-in of 100 000 followed by 500 000 Markov Chain Monte Carlo (MCMC) steps for each run. The geographic location of samples was not used in the clustering analysis. Results across each run were summarised to infer the optimal value of *K* using the method of Evanno et al. [17], as implemented in STRUCTURE HARVESTER web v. 0.6.93 [16], processed using CLUMPP v. 1.1.2 [28] to determine the optimal alignment of each of the ten iterations, and visualised with DISTRUCT v. 1.1 [49].

To ascertain the likelihood of recent bottlenecks due to severe range contraction and subsequent founder effects, the program BOTTLENECK v. 1.2.02 [47] was employed to investigate whether the genetic status of current small fragmented populations was outside mutation-drift equilibrium and if the excess of heterozygosity expected after a bottleneck was significant. We tested for significant departure from equilibrium of each population using Sign and Wilcoxon’s signed rank tests conducted under the assumptions of the intermediate two-phased model (TPM), due to its suitability for microsatellite data [47].

## Availability of supporting data

All data supporting the conclusions to this study can be found within the manuscript and its additional files.

## Additional files

Additional file 1: Table S2. Summary of AMOVA (PhiPT) for the 218 individuals sampled from seven populations of *F. picrosperma*. df, degrees of freedom; SS, sum of squared deviations; MS, mean sum of squared deviations; Est. Var., estimates of variances; %, percentage of variance. (DOCX 12 kb) Additional file 2: Figure S1. Scree plot of eigenvalues from principal coordinates analysis (PCoA) of *F. picrosperma* individuals using genetic distance matrices. Scree plot of eigenvalues of components 1 to 25 from the principal component analysis. (PDF 10 kb) Additional file 3: Table S1. Genetically differentiated groups of populations as determined using Bayesian genetic clustering analysis. STRUCTURE analysis indicated that ln likelihoods of the data plateaued quickly from *K* = 3 to *K* = 4. *K* = 3 was selected as the best estimate of the number of genetic clusters following implementation of the Evanno method [17]. (DOCX 32 kb)
