# Supplementary material for: Population genetic analysis of a medicinally significant Australian rainforest tree, Fontainea picrosperma C.T. White (Euphorbiaceae): biogeographic patterns and implications for species domestication and plantation establishment
Source: BMC Plant Biol. 2016 Feb 29;16:57. doi: 10.1186/s12870-016-0743-2 (PMC4772518; doi:10.1186/s12870-016-0743-2)
Supplement: Additional file 1: Table S2. — Summary of AMOVA (PhiPT) for the 218 individuals sampled from seven populations of F. picrosperma. df, degrees of freedom; SS, sum of squared deviations; MS, mean sum of squared deviations; Est. Var., estimates of variances; %, percentage of variance. (DOCX 12 kb) [file 12870_2016_743_MOESM1_ESM.docx]

**Table S2: Summary of AMOVA (PhiPT) for the 218 individuals sampled from seven populations of *F. picrosperma*.** df, degrees of freedom; SS, sum of squared deviations; MS, mean sum of squared deviations; Est. Var., estimates of variances; %, percentage of variance.

|  | df | SS | MS | Est. Var. | % |
| --- | --- | --- | --- | --- | --- |
| Among Pops | 6 | 337.396 | 56.233 | 1.727 | 25 |
| Within Pops | 211 | 1103.737 | 5.231 | 5.231 | 75 |
| Total | 217 | 1441.133 |  | 6.958 | 100 |
