# Supplementary figures and images for: Population genetic analysis of a medicinally significant Australian rainforest tree, Fontainea picrosperma C.T. White (Euphorbiaceae): biogeographic patterns and implications for species domestication and plantation establishment
Source: BMC Plant Biol. 2016 Feb 29;16:57. doi: 10.1186/s12870-016-0743-2 (PMC4772518; doi:10.1186/s12870-016-0743-2)

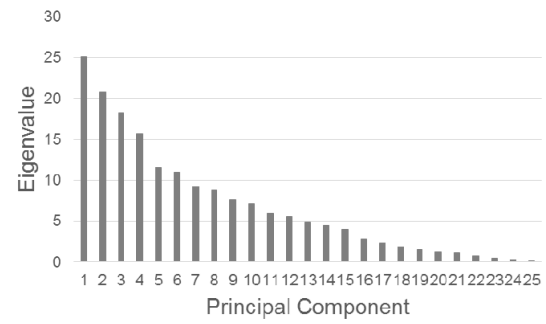

Supplement: Additional file 2: Figure S1. — Scree plot of eigenvalues from principal coordinates analysis (PCoA) of F. picrosperma individuals using genetic distance matrices. Scree plot of eigenvalues of components 1 to 25 from the principal component analysis. (PDF 10 kb) [file 12870_2016_743_MOESM2_ESM.pdf]
