# Supplementary material for: Population genetic analysis of a medicinally significant Australian rainforest tree, Fontainea picrosperma C.T. White (Euphorbiaceae): biogeographic patterns and implications for species domestication and plantation establishment
Source: BMC Plant Biol. 2016 Feb 29;16:57. doi: 10.1186/s12870-016-0743-2 (PMC4772518; doi:10.1186/s12870-016-0743-2)

**Table S1: Genetically differentiated groups of populations as determined using Bayesian genetic clustering analysis.** STRUCTURE analysis indicated that ln likelihoods of the data plateaued quickly from K=3 to K=4. K=3 was selected as the best estimate of the number of genetic clusters following implementation of the Evanno method (Evanno et al. 2005).


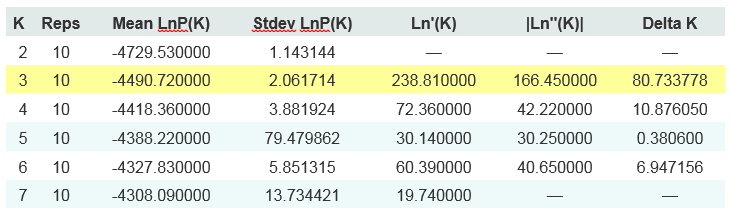

Supplement: Additional file 3: Table S1. — Genetically differentiated groups of populations as determined using Bayesian genetic clustering analysis. STRUCTURE analysis indicated that ln likelihoods of the data plateaued quickly from K = 3 to K = 4. K = 3 was selected as the best estimate of the number of genetic clusters following implementation of the Evanno method [17]. (DOCX 32 kb) [file 12870_2016_743_MOESM3_ESM.docx]
